# Isolation and culture of fibroblasts from endoscopic duodenal biopsies of celiac patients

**DOI:** 10.1186/1479-5876-7-40

**Published:** 2009-06-04

**Authors:** Leda Roncoroni, Luca Elli, Luisa Doneda, Luca Piodi, Michele M Ciulla, Roberta Paliotti, Maria Teresa Bardella

**Affiliations:** 1 Center for Prevention and Diagnosis of Celiac Disease, Fondazione IRCCS Ospedale Maggiore Policlinico, Mangiagalli e Regina Elena, Milan, Italy; 2 Department of Medical Sciences, University of Milan, Italy; 3 Department of Biology and Genetic for the Health Sciences, University of Milan, Italy; 4 Gastroenterology II, Fondazione IRCCS Ospedale Maggiore Policlinico, Mangiagalli e Regina Elena, Milan, Italy; 5 Cardiothoracic Department, Institute of Cardiovascular Medicine, Center of Clinical Physiology and Hypertension, Laboratory of Clinical Informatics and Cardiovascular Imaging, University of Milan, Italy

## Abstract

**Background:**

Fibroblasts are actually considered pivotal in inflammation and tissue remodelling process and for these reasons they are involved in the pathogenesis of autoimmune disorders such as celiac disease. Investigations to define the role of fibroblasts in celiac diseases are obstructed by the absence of specific models. Our objective is to isolate and culture primary fibroblasts from endoscopic duodenal biopsies of celiac and non-celiac subjects, to analyze their growth patterns and the morphometric characteristics.

**Methods:**

60 duodenal bioptic specimens from 20 celiac patients and 114 from 38 non-celiac subjects were mechanically chopped and enzymatically digested in order to obtain primary cell cultures. Growth patterns, karyotype (Q-banding analysis), expression of typing proteins (fibroblast surface protein and cytokeratin 20) and morphometric parameters (diameters and their ratio, perimeter, area and perimeter/area ratio at computerised image analysis) were investigated on cultured cells.

**Results:**

Primary cells were successfully cultured in 78% of the collected duodenal biopsies. Cultured cells, expressing the fibroblast surface protein, were negative for cytokeratine 20 and maintained a normal kariotype. Cells grew slowly without differences between the celiac and the non celiac group. Morphometric analysis of celiac fibroblasts revealed significantly increased dimensions, with a preserved diameters ratio, and a reduced perimeter/area ratio.

**Conclusion:**

For the first time this study demonstrates the feasibility of culturing primary fibroblast cell from endoscopic duodenal biopsies in celiac and non-celiac subjects, opening a new window of opportunity in studies intended to establish the role of fibroblasts as a possible partaker in the pathogenesis of the celiac mucosal damage.

## Introduction

Celiac disease (CD), the most common chronic enteropathy in Western countries, affects genetically predisposed subjects carrying HLA-DQ2 or DQ8 after the ingestion of prolamins (gliadins) present in wheat, rye and barley; Although the CD pathogenesis is largely unknown, it is considered an autoimmune disease due to the abnormal activation of immune system and the presence of autoantibodies [1,2]. Different cell types (enterocytes, lymphocytes B and T, macrophages, dendritic and mesenchymal cells) participate in the development of the CD small bowel mucosal damage, characterised by lymphocytic infiltration and villous architectural rearrangement [3,4], and in particular fibroblasts (FBs) seem to have a central role due to their involvement in inflammatory mechanisms and tissue remodelling. The traditional idea of FBs has been evolved from merely extracellular matrix (ECM) producers to transducers of complex environmental stimuli, supporting their central role in the pathogenesis of different human pathologies such as fibrotic diseases, infections, tumors and autoimmune disorders [5-7]. The biological functions exerted by FBs are linked to the secretion of enzymes (metalloproteases-MMPs, tissue inhibitor of metalloprotease-TIMP, transglutaminase type 2-TG2) [8-12], cytokines and chemokines (transforming growth factor β-TGFβ, tumor necrosis factor α-TNFα, interferon γ-IFNγ, interleukins-ILs, monocyte and granulocyte chemotactic proteins, RANTES) [13-17], prostaglandines [18], proteins of the extracellular matrix (ECM) [19]. Moreover, they take part in the intercellular network through the presence on their cell membrane and in the intracellular space of different types of receptors (receptors for E series of prostaglandins, insulin-like growth factor 1 receptor, 5-HT receptor-associated proteins, nuclear fibroblast growth factor receptor-1 and cytokine receptors) [5,20-22]. Researches about the involvement of FBs in CD are actually obstructed by the absence of a specific models; we therefore aimed this study to isolate and culture primary FBs from endoscopic duodenal biopsies of CD and non-CD subjects, to analyze the growth patterns of the cultures and to compare the basic morphometric characteristics of FBs.

## Methods

### Patients

From September 2006 to January 2008, 58 consecutive subjects undergoing EGDS and agreeing to the study, were enrolled. Twenty CD (9 males and 11 females, median age 41, range 25–55), 11 (5 males and 6 females, median age 40, range 25–43) following a gluten containing diet and 9 (4 males and 5 female, median age 48, range 30–55) following a gluten free diet (GFD) (median years on a GFD 7, range 1–20), and 38 non-CD (18 males and 20 females, median age 45, range 24–56) patients. CD diagnosis was based on the presence of the serological markers anti-tissue-transglutaminase (ELISA or radioimmunoassay tests) and/or anti-endomysium (immunofluorescence technique) IgA antibodies and a Marsh-Oberhuber III duodenal histology [23,24]. Marsh-Oberhuber grading was used to evaluate duodenal histology [24]. Adherence to the GFD was based on negativization of serological CD markers. Non-CD group was composed by dyspeptic subjects without endoscopic or histological lesions, not referring other autoimmune or intestinal diseases.

From each patient 3 duodenal biopsies were taken for a total of 60 CD and 114 non-CD specimens.

The study was approved by the ethical committee of the "Fondazione IRCCS Ospedale Maggiore Policlinico, Mangiagalli e Regina Elena – Milano".

### Duodenal specimens and cell cultures

During EGDS (Olympus endoscopes, Japan), duodenal tissue specimens were taken by the use of standard endoscopic forceps (Boston Scientific, USA); they were rapidly dipped into sterile tubes (Becton and Dickinson, Italy) containing 3 mL of medium composed by DMEM (GIBCO, Italy) supplemented with 4% penicillin 100 U/mL-streptomycin 100 μg/mL (GIBCO, Italy) during the transport from the endoscopy room to the cell culture laboratory (approximately 15 minutes).

At the laboratory, biopsy samples were gently washed three-times with 4 mL of PBS without Ca^2 ^and Mg^2 ^(GIBCO, Italy), moved into a tissue culture dish (60 × 15 mm) (Corning, Italy) and finely chopped with a disposable surgery knife for approximately 10 minutes; samples were incubated in Ham's F12 medium (GIBCO, Italy), containing liberase blendzyme 2 (1.4 W/mL) (Roche, Italy) at 37°C for three hours in CO_2_. Digestion terminated by centrifugation (1000 × g for 5 minutes) and the obtained tissutal pieces and floating cells were seeded onto the cell culture Petri dishes (35 × 10 mm) (Nunc, Italy) in 2 mL of medium composed by Ham's F-12 (GIBCO, Italy), foetal bovine serum 10% (GIBCO, Italy) supplemented with 4% penicillin 100 U/mL-streptomycin 100 μg/mL (GIBCO, Italy), covered with cover glasses and incubated at 37°C in 5% CO_2_. The medium was replaced every 6 days.

After first passage cells were passed in T25 flasks (Corning, Italy) and pooled for each patient; passages were enzymatically performed by a 1:2 split. Cells at passage 3 were used for the studies.

Supplemental 10 bioptic specimens from CD and non-CD group were rapidly dipped into 2 mL cryovials (Corning, Italy), nitrogen frozen and successively weighted (Gibertini E42S, Italy).

Mycoplasma contamination was routinely checked and excluded by mean of Hoechst method [25].

**Figure 1:**
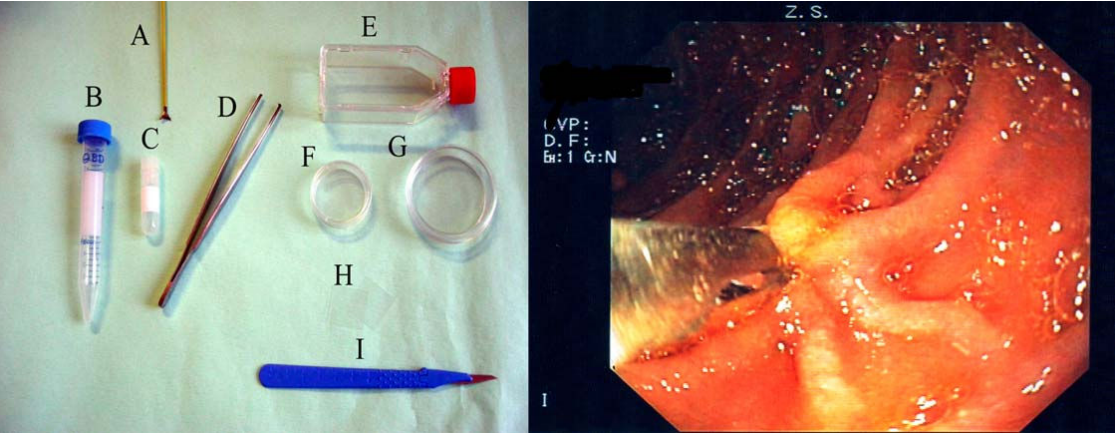
**Left panel: disposable materials used in primary fibroblast cultures; A endoscopic forceps, B and C tubes, D laboratory forceps, E T25 flask, F and G Petri dishes, H cover glasses, I surgical knife.** Right Panel: duodenal endoscopic biopsy procedure.

Cell cultures were observed by phase contrast microscopy to verify growth, and viability was routinely checked by a trypan blue-dye exclusion assay (Sigma, Italy). Cultures showing a viability > 95% were used. Materials used are shown in figure 1.

### Immunocytochemistry

FBs were typed by using a conventional marker (FB surface protein-FSP, monoclonal anti-human FSP, Clone 1B10; Sigma, Italy) and epithelial types were carefully excluded performing cytokeratine analysis (anti-human Cytokeratin 20; Sigma, Italy); primary antibodies were used at the manufacturer recommended dilutions. Cells were seeded onto 24 well plates at a concentration of 20.000 cells/plate; after 48 hours they were washed twice in PBS and fixed with 3.7% formaldehyde in PBS for 15 minutes at room temperature (RT). Fixed cells were permeabilised with 0.1% triton X-100 (Sigma, Italy) in PBS for 15 minutes at RT. Non specific binding of secondary antibody was blocked by incubation with normal foetal serum for 30 minutes at RT. After immunostaining cells were rinsed with PBS and incubated with fluorochrome conjugated secondary antibody for 45 minutes at RT, according to donor species of the primary antibodies. PBS was used as the negative control in place of the primary antibody. Counterstaining was performed using DAPI; the glass coverslip was mounted on glass slides with prolong gold antifade reagent (Invitrogen, Italy). Images were obtained by fluorescence microscope (Leica, Italy).

### Q-Banding

Cells in log phase were cultured with 50 μL of colchicine for 4 hours and mitotic cells were gently blown with a pipette. Cells were centrifuged at 235 × g for 10 minutes and the supernatant fluid removed. KCl at 37°C (0.075 M) was added to the cells and the mixture incubated at 37°C for 30 minutes. Cells were then fixed with 3:1 methanol/acetic acid and cell suspensions dropped onto slides, air dried and stained with quinacrine stain for 20 minutes. Slides were observed with oil immersion at fluorescence microscopy (Leica, Italy) [26].

### Image capture and morphometric analysis

Culture growth and FBs morphometric analysis were performed on low-power fields (10× magnification) with a microscope (Nikon, Italy) coupled with a digital CCD camera. Images were stored on a personal computer (Power Mac G4, 1.25 GHz, 512 MB RAM, Apple, Cupertino, CA) in TIFF format. Stored images were analyzed in the Laboratory of Clinical Informatics and Cardiovascular Imaging, University of Milan by a single experienced reader blinded to image sequence and assignment. Analysis algorithms were developed as a set of macros executed with NIH Image, an integrated image-processing software distributed on a freeware basis by the National Institutes of Health (Bethesda, USA). Before the analysis, an automated threshold process was performed on the images to minimize the influence of light variation in the microscope field and in the operator subjective settings. This process cuts off any object below the minimum signal intensity. FBs were recognized on the basis of their sizes and intensity signal by using a cell count algorithm that draws a region of interest (ROI) around each discrete object whithin the image. The minimum sizes in pixels of the objects to be included in the count was previously defined by accurately measuring 12 representative FBs. Objects below the minimum size were not included in the count, cells closely adjacent to each other (touching edges) were excluded. The culture growth was determined on days 12, 20, and 30 by counting the number of recognized FBs over the area (microscopic field). The morphometric evaluation included the major orthogonal diameters and their ratio, as index of circularity, the perimeter, the area, and their ratio, as index of complexity.

### Statistical analysis

Data were expressed as mean ± standard deviation (SD) or median and range. A comparison of the morphometric data obtained from culture of CD and non-CD subjects was done using one way ANOVA. All statistical analysis was performed using statistical computer software (SPSS 13, SPSS, USA). A p value of 0.05 was considered significant.

## Results

Sixty CD and 114 non-CD duodenal bioptic specimens were successfully obtained from EGDSs and from each biopsy a similar amount of tissue weight was processed from CD and non-CD (54.9 mg ± 6.4 vs 56.7 mg ± 4.8; respectively; p = ns). All the CD patients on a gluten containing diet had villous atrophy (type 3 lesion); among the CD patients on GFD, all serologically negative, histology showed type 0, 1, 2 and 3a in 2 cases each and type 3b in 1 case. Non CD patients were all classified as type 0.

**Figure 2:**
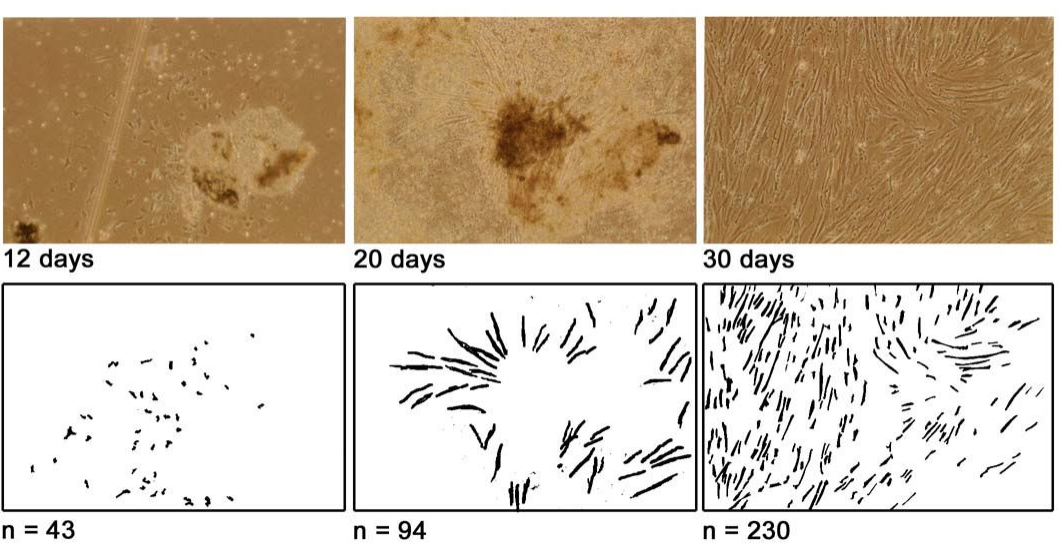
**Cellular growth from a chopped and enzymatically digested fragment of endoscopic duodenal biopsy at different times after seeding as visualised at microscopy (10 × magnification, upper panels) and after computer image analysis skeletonizing objects compatible with cells (fibroblasts) evidencing growth pattern radially spreading from the tissue sample**.

After 8–12 days of culture, FB-like cells growing radially from the chopped and enzymatically digested bioptic pieces were observed; their lateral spreading increased dimensionally during the culture and the first passage was performed at day 30 (range 25–35) with confluent cells (Figure 2 upper panels). Next passages were performed monthly (range 25–40 days). Primary cultures survived for at least six passages and usually died after 180 days (range 170–200) of culture. The duplication time was about 8 days both for CD and non-CD cells (Figure 2 lower panels), with no statistical differences.

Out of the 174 duodenal specimens, 135 (78%; 45 CD, 90 non-CD) completed the entire cycle of culture. The major reasons of unsuccessful were bacterial contamination (18%) and insufficient bioptic material (4%), equally distributed between the 2 groups (data not shown). Sex, age, clinical and dietary status in the CD group (patients following a gluten-containing or a gluten-free diet) did not influence the successful rate or growth indexes of cell cultures.

**Figure 3:**
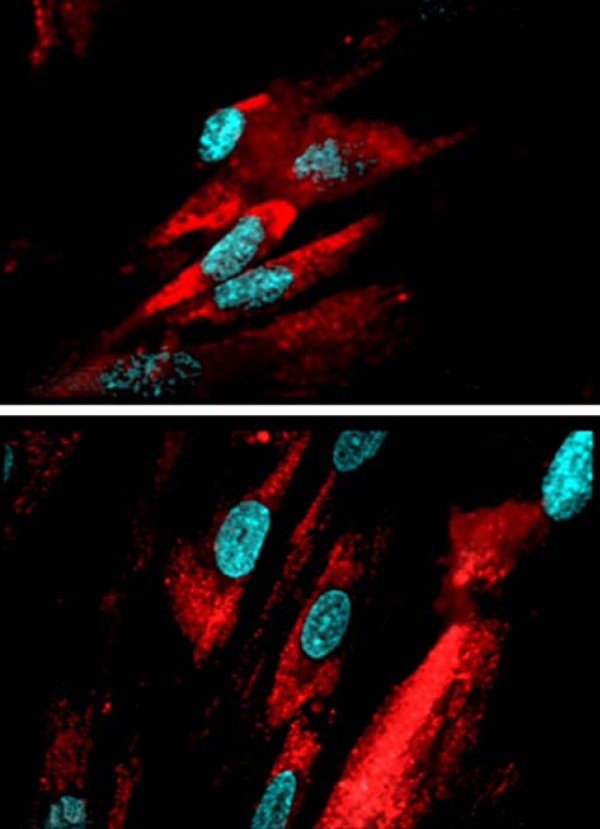
**Fibroblast surface protein immunocytochemistry of primary cells from duodenal endoscopic biopsies from celiac (upper panel) and non celiac (lower panel) patients; DAPI counterstained cellular nuclei**.

Immunocytochemistry was positive for FSP and negative for cytokeratin 20 in all the cultured and examined cells (Figure 3).

Q-banding analysis of FBs from CD and non-CD subjects demonstrated a normal and stable karyotype (data not shown).

**Figure 4:**
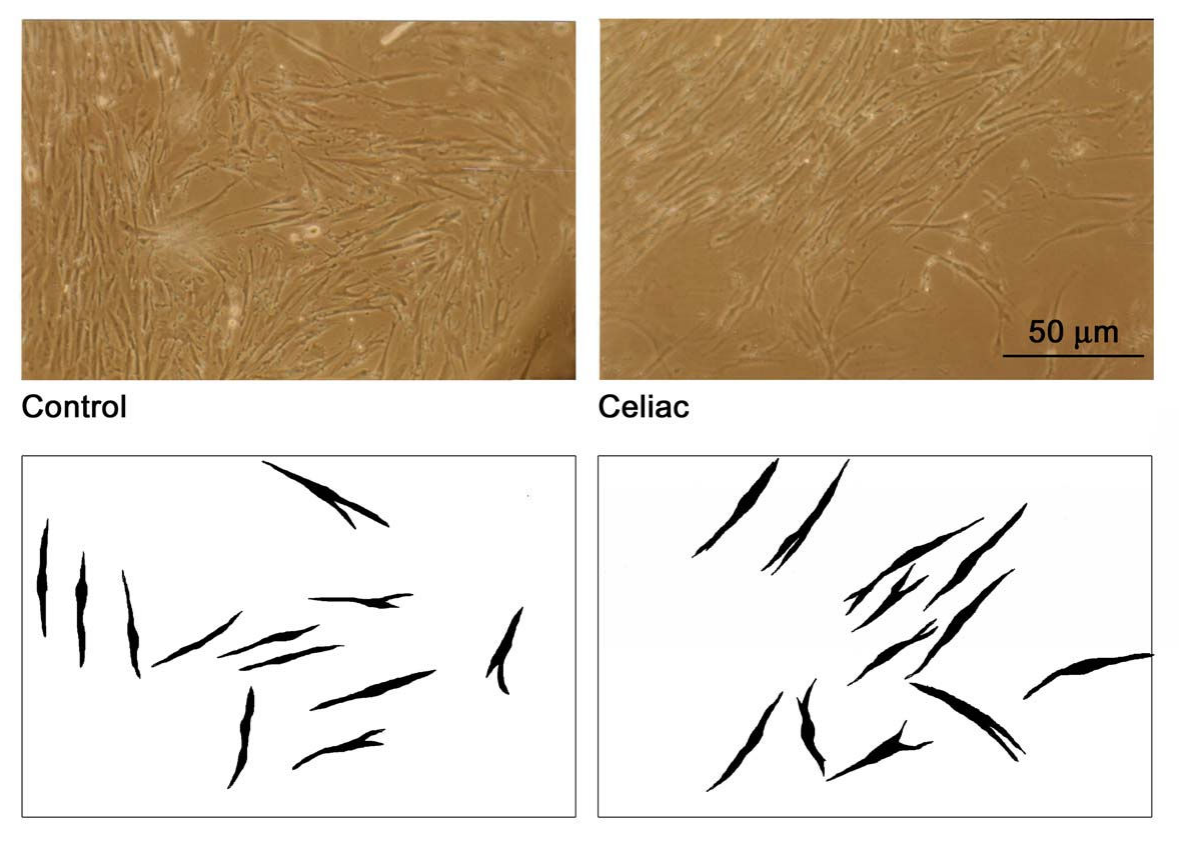
**Contrast microscopy images of celiac and non celiac (control) fibroblasts at the third passage (upper panel) and skeletonized computer image analysis used for morphometric measurements**.

Morphometrical analysis performed on CD and non-CD FBs images obtained at the same day of culture (Figure 4) showed some significant differences; in particular, CD FBs were greater, with a longer diameter and perimeter and the area was wider even if the circularity index was similar; on the other side the complexity index was decreased, suggesting a change in the cellular membrane-cytoplasm ratio (Table 1). In particular dietary status and the Marsh-Oberhuber histological grading of CD patients did not influenced morphometric parameters.

**Table 1:** Morphometrical characteristics of cultured fibroblasts

**Parameter**	**Non-CD**	**CD**	***p***
Feret Diameter (μm)	40.15 ± 5.15	46.79 ± 8.09	0.025
Perimeter (μm)	91.93 ± 15.43	113.30 ± 19.11	0.0064
Area (μm^2^)	88.79 ± 22.52	122.86 ± 19.11	0.0018
Circularity index	0.13 ± 0.02	0.12 ± 0.02	ns
Complexity index (μm^-1^)	1.06 ± 0.16	0.93 ± 0.11	0.033

Feret Diameter: longest axis; Circularity index = Feret Diameter/Short axis length; Complexity index = perimeter/area.

A p < 0.05 was considered significant; ns = not significant

## Discussion

FBs are known to be involved in inflammation and tissue remodelling, and they play a pivotal role in CD [27]. Unfortunately, till now no experiences have been reported on culturing primary cells obtained from endoscopic duodenal biopsies, the most reliable source of primary intestinal cells. In this study, for the first time, we describe a suitable technique to obtain long-standing primary human FB cultures from endoscopic biopsies.

In the absence of standardised systems, we based FBs extraction method on those used for cultures from surgical pieces, muscle and skin tissue samples [28]. Differently from these specimens, intestinal endoscopic biopsies contain a small amount of a soft tissue and have an important bacterial contamination caused by the common intestinal flora, the manual management of the endoscope and endoscopic forceps, and their passage through the endoscopic channel together with the patients' gastric juice and saliva. For these reasons we used a higher dose of antibiotics and the proteolytic cocktail of enzymes liberase, rather than the traditional collagenase cocktails that are known to contain endotoxin and exert cytotoxic effects on primary cultures with an increase of lipidic intracellular droplets [29]. Liberase is a blend of highly purified enzymes used to improve the isolation and cultures derived from small tissutal specimens, not suitable for mechanical isolation [30-32].

In our study 96% of the endoscopic samples resulted adequate to obtain cells without differences between the specimens obtained from CD patients and those from control subjects. All the cultured cells were FBs with normal karyotype, as demonstrated by the FSP positivity, cytokeratine 20 negativity, and the Q banding analysis.

The successful rate of cell cultures was 78%, higher than those obtained from transbronchial lung endoscopic biopsies (successful rate 54%) [33], the only available to make a comparison, since there are no data on mesenchymal cell extraction from endoscopic duodenal biopsies. This success rate was not affected by other possible covariates such as the clinical and demographic characteristics of patients suggesting that the stabilization of cell culture is almost technique-dependent. We judged our rate of success acceptable, taking into consideration the technical difficulties and the bacterial load, the most important cause of withdrawal (18%).

In CD, FBs are known to take part in the development of the intestinal damage (villous atrophy) regulating the deposition, degradation and remodeling of the ECM through the secretion of collagen, MMPs, TIMPs, and TG2, usually altered in CD intestinal mucosa [34-37]. Moreover, FBs cooperate in the establishment of the CD immunomediated reaction and enterocyte differentiation through the secretion of TGFβ and as a target of the celiac autoantibodies, which finally influence their cell cycle inducing an S phase shifting and the TGFβ secretion [38,39]. However, these observations are derived from studies on immortalised cell lines (NIH 3T3 and IMR90 FB), human umbilical chord-derived FBs and cultured duodenal biopsies. Although these techniques provide important high-technology resources, they have some constrains: immortalised cell lines are important to study cytotoxic effects in a simplified protein/xenobiotic-cell microenvironment, but they are not disease-specific [40-42]; cultured duodenal biopsies are a human and disease-specific technique, but they survive in laboratory setting for a maximum of 72 hours, conditioning the study of chronic long-term mechanisms [43]. Furthermore, there are no suitable animal models for investigating CD: the Irish setter dog gluten-induced enteropathy and the rhesus macaques non-infectious diarrhoea are the most CD-specific, but they are expensive, not accessible to a high number of researchers, non-human and involve ethical aspects [44,45].

Thus, the cultures of primary CD FBs represent an important research aid, easy to obtain because all CD patients undergo to EGDS.

By using a morphometric approach, based on five parameters, we found significant differences between cultured control and celiac FBs; in particular, celiac FBs were substantially longer and wider, with a preserved circularity but a reduced complexity index (perimeter/area ratio) if compared with control FBs. These characteristics are specific of celiac FBs independently by the dietary status of the patients and the Marsh-Hoberhuber histologic grading, suggesting a "permanent" alteration. Since it is well known that shape and size of cells are the result of the spatial arrangement of the microtubule cytoskeleton and are closely related to cell function, we cannot exclude that these differences reflect, at least in part, a different functional state and/or a phenotype. It is noteworthy that the reduced perimeter/area ratio suggests for cultured celiac FBs a lower shape complexity, a parameter that normally is under tight control to ensure a normal cell architecture and tissue pattern [46]. At this regard it should be noticed that standard *in vitro *cell culture models do not represent the *in vivo *structure, nonetheless the differences observed between control and celiac groups were obtained in the same culture conditions.

## Conclusion

Primary cell cultures from duodenal endoscopic biopsies provide human disease-specific material and are easily suitable in all patients; in fact previous studies using primary cultures from the GI tract were performed only by sampling surgical pieces, thus excluding non-surgical patients, that are known to represent the majority of affected ones. The method of cell culture here described could help in the establishment of novel experiments to study the role of FBs in the pathogenesis of the mucosal damage and to test new therapies alternative to the gluten-free diet. In this context, endoscopy can revalue its role from a simple diagnostic and therapeutic method to a determinant technique in basic translational research.

## Competing interests

The authors declare that they have no competing interests.

## Authors' contributions

Conception and Design: LR, LE.

Data Analysis: MC, RP.

Drafting the article: LD, LP.

Critical Revision and Final Approval: MTB.
